# The expansion of 8-year medical training programs in China: a status report

**DOI:** 10.3402/meo.v20.25890

**Published:** 2015-02-06

**Authors:** Zhao Wang, Zhuo Yin, Yong-bao Wei, Long-fei Liu, Jin-rui Yang

**Affiliations:** 1 Department of Urology, The Second Xiangya Hospital, Central South University, Changsha, China; 2 Department of Urology, Xiangya Hospital, Central South University, Changsha, China

**Keywords:** medical education, China, cultivating objective, teaching model

## Abstract

Instituted in major medical programs only within the past decade, the advent of an ‘expanded’ 8-year medical curriculum reflects a major reformation of how physicians are trained in China. Although much remains to be done, including the refinement of associated learning objectives, instructional models, and teaching pedagogies, movement toward a longer, more standardized training framework represents a marked transition for Chinese medical practice. This article highlights the current status and anticipated future of these emerging 8-year medical training programs in modern-day China.

With the continued economic development and growth in China, the expectations from the country's health care system also have risen. Because any effective public health system relies heavily on the quality and supply of medical providers, a historic series of related reforms in China are now occurring (1). One such change, intended to meet these growing societal demands, is the continued transition to an 8-year curricular training model for future physicians and medical scientists (2).

The 8-year curricular framework, common in many parts of the industrialized world, was first implemented in Peking Union Medical College (PUMC) – which was founded in 1917 by the Rockefeller Foundation (2). Indeed, prior to 2001, PUMC was the only Chinese medical college to offer an ‘extended’ medical training plan leading to the Doctor of Medicine (MD) degree. In 2001, Peking University Health Science Center (PUHSC) received approval from the Ministry of Education to pilot an 8-year program, followed shortly thereafter by five additional comprehensive universities and four military medical schools (3). Presently, there are 18 leading medical schools in China boasting of medical education programs with an 8-year curriculum (4, 5).

## Proposals and policies

In 2009, the Chinese government announced major comprehensive health reforms aimed at establishing a system of basic health care to provide universal coverage to its citizenry – prompting the establishment of 3-, 5-, and 8-year medical programs leading to diploma, bachelor's, and doctoral degrees, respectively (6). The Ministries of Education and Health have since issued accreditation standards for the 5-year programs, which are anticipated to be the primary pathway for most medical doctors (6). The longer 8-year programs, in contrast, are designed to cultivate professional physicians. However, although resource concerns have restricted these expanded curricula to the nation's leading medical schools, individual programs have been given considerable autonomy in crafting their curricula. As a result, the emerging models have been surprisingly diverse.

In 2009, medical educators drafted clinical teaching objectives and basic requirements for 8-year programs based on minimum essential standards, basic requirements, and lessons gleaned from the PUMC experience (7). The primary goal of such programs, it was decided, was to produce master diagnosticians and physicians in the treatment and prevention of common diseases. Toward this end, different schools were allowed to adopt different foci: some schools, such as PUHSC and Sichuan University, emphasize clinical practice, whereas others (e.g., Tsinghua University and Shanghai Jiao Tong University) are oriented toward clinical science (8, 9). Still others, such as the Central South University (CSU) and Sun Yat-sen University, may integrate multiple aspects of medicine, including basic clinical skills, scientific research, and critical thinking (10, 11).

## Prevailing models: admission and switch

In China, there presently exist two basic types of 8-year medical education programs: the ‘8-year consistent’ model and the ‘4+4’ model. The former is the prevailing educational model in which students pass the National College Entrance Examination (NCEE) before being admitted to medical school from high school. Only two schools offer ‘4+4’ programs: Shanghai Jiao Tong University and Zhejiang University. Here, students are admitted to medical school with baccalaureate degrees from leading Chinese universities – similar to many Western medical programs.

To ensure optimal performance in these extended 8-year programs, students' progress is carefully monitored, and outstanding students may switch to this program from other majors such as chemistry, physics, and biology. These academic guidelines vary by program: during year two at CSU, for example, the poorest performers may be demoted to a 4-year track of any major, whereas underperforming students in years 3–6 may be relegated to a 5-year track.

## Training tracks

In both 8-year curricular models, contents are separated into premedical, basic medical, clinical medical, clerkship, and research training. Here, again, the exact allocation of time is program specific or, to a lesser extent, model specific. For instance, PUMC dedicates roughly 2.5 years for premedical education, 1.5 years for basic medical education, 4 years for clinical training, and 8 months for research. Students complete premedical training at Tsinghua University – concentrating on biology and the social sciences. Basic and clinical education is undertaken at PUMC. After finishing these courses and clerkships, students get research experience (also at PUMC) in areas of personal interest. Provided they pass their dissertations, students of 8-year medical programs will be awarded a MD degree (2). Other ‘8-year consistent’ programs may split students into different colleges to further study science (e.g., chemistry, physics, etc.) at CSU during the 2-year premedical phase. However, premedical content at PUHSC is condensed into a single year.

In the ‘4+4’ model at Shanghai Jiao Tong University, the training track is divided into four 1-year stages: 1) basic medicial education, 2) clinical medical education, 3) clerkship training, and 4) research training.

## Teaching models

The dominant pedagogy in most Chinese medical schools is traditional, discipline-based lecture, and involves coursework in anatomy, physiology, pathology, diagnostics, internal medicine, surgery, pediatrics, obstetrics, and gynecology. In recent years, approaches based on integrated organ systems have been incrementally introduced in some Chinese medical schools (12).

## Perspectives on educational reformation

Although the 8-year medical curriculum has existed at PUMC for nearly a century, it has become widely implemented throughout China only in the past decade. Because it lacks the necessary structure and detail of more mature programs, we offer the following observations and recommendations.

First, detailed learning objectives must be established which span all stages of the 8-year curricula. All objectives need not be identical and should reflect individual programs' unique curricular thrusts; however, they should be readily measureable.

Second, although program emphases may vary, standardization of other key processes (e.g., degree granting requirements, residency training, accreditation) is highly desirable. As a specific example, mentoring programs should be established and evaluated across all schools (13). Similarly, a rigorous system should be established to assess students' abilities and competencies prior to determining postgraduate education and training tracks. Faculty and curricular evaluation should also be undertaken to monitor and ensure that students receive a comparable, quality learning experience.

Third, because only the top medical schools in China are eligible to enroll students directly from NCEE or baccalaureate studies directly into an 8-year medical training program, it is estimated that the number of medical schools offering such curricula needs to increase dramatically (5). As stated, a valid and defensible system of accreditation is also needed to gauge the qualifications of prospective schools wishing to offer expanded 8-year training programs.

Fourth, investigations should be undertaken, and subsequent adjustments made, to adapt an integrated instructional approach based either on organ systems or discipline, as per the resources and circumstances of individual schools.

Finally, compared with 5-year training models, international learner exchanges are much more common in 8-year training programs featuring clinical rotations, lab rotations, and summer courses (14). Because these experiences broaden the cultural horizons students, as well as provide necessary financial support and learning certification, they should be encouraged.

## Conclusions

Although variations in 8-year medical training curricula represent the current standard in many areas of the world, they are relatively novel in China and constitute a developing ‘work in progress’. Continued changes and reforms should strive to further these efforts by developing, among other things, formal educational plans with clear, measurable, and valid learning objectives. Additional research into effective teaching pedagogies is also warranted to optimize fit within China's emerging but diverse 8-year medical training programs.
